# Exploring the Contrasts and Similarities of Dengue and SARS-CoV-2 Infections During the COVID-19 Era

**DOI:** 10.3390/ijms252111624

**Published:** 2024-10-29

**Authors:** Alexis Hipólito García, Juan Bautista De Sanctis

**Affiliations:** 1 Institute of Immunology Nicolás Enrique Bianco, Faculty of Medicine, Universidad Central de Venezuela, Caracas 1050, Venezuela; 2 Institute of Molecular and Translational Medicine, Faculty of Medicine and Dentistry, Palacky University, Hněvotínská 1333/5, 77900 Olomouc, Czech Republic; 3 Czech Advanced Technology and Research Institute, Palacky University, 77900 Olomouc, Czech Republic

**Keywords:** dengue infection, SARS-CoV-2 infection, COVID-19, platelets, antibodies, cytokine storm, antibody-dependent enhancement (ADE)

## Abstract

Extensive research has been conducted on the SARS-CoV-2 virus in association with various infectious diseases to understand the pathophysiology of the infection and potential co-infections. In tropical countries, exposure to local viruses may alter the course of SARS-CoV-2 infection and coinfection. Notably, only a portion of the antibodies produced against SARS-CoV-2 proteins demonstrate neutralizing properties, and the immune response following natural infection tends to be temporary. In contrast, long-lasting IgG antibodies are common after dengue virus infections. In cases where preexisting antibodies from an initial dengue virus infection bind to a different dengue serotype during a subsequent infection, there is a potential for antibody-dependent enhancement (ADE) and the formation of immune complexes associated with disease severity. Both SARS-CoV-2 and dengue infections can result in immunodeficiency. Viral proteins of both viruses interfere with the host’s IFN-I signaling. Additionally, a cytokine storm can occur after viral infection, impairing a proper response, and autoantibodies against a wide array of proteins can appear during convalescence. Most of the reported autoantibodies are typically short-lived. Vaccines against both viruses alter the immune response, affecting the course of viral infection and enhancing clearance. A comprehensive analysis of both viral infections and pathogenicity is revisited to prevent infection, severity, and mortality.

## 1. Introduction

Dengue virus infection is prevalent in tropical regions, especially during the rainy season. It is transmitted to humans through the bite of infected mosquitoes, posing a risk to approximately half of the global population and resulting in an estimated 100–400 million infections annually with around 7500 deaths [1]. However, this may be an underestimation [1]. In contrast, the SARS-CoV-2 pandemic has impacted over 750 million individuals worldwide (WHO) since 2019, with a mortality rate close to 1% [2]. 

During the SARS-CoV-2 pandemic, there was a surge in dengue and severe dengue cases in high-risk areas, leading to various hypotheses. Two main hypotheses have been proposed to explain the potential increase in severe dengue cases following SARS-CoV-2 infection [3,4,5]. One is related to viral protein similarities and the antibodies generated to possible common epitopes, which will increase viral immunopathology, especially in dengue infection. The other assumption refers to immune deficiency after the viral infection that predisposes new viral infections. However, the reported data have yielded contradictory findings [5,6], potentially attributed to variations in experimental trials, screenings, and affected populations. Therefore, this review aims to comprehensively examine the viral physiopathology in dengue and SARS-CoV-2 infections, identifying their similarities, differences, and the potential impact of co-infection or previous infection on disease resolution.

### 1.1. Dengue Virus (DENV)

Dengue is caused by the dengue virus (DENV), a single positive-stranded RNA virus of the Flaviviridae family, transmitted to humans through the bite of infected female mosquitoes, mainly the Aedes aegypti mosquito. Other species of the genus Aedes can also be transmitters; their contribution is usually less than that of Aedes aegypti, a vector found predominantly in tropical and subtropical areas of the planet. Several tropical regions have a hyperendemic form of dengue infection, with different forms of dengue fever [7,8,9]. The causative microorganism of dengue is a virus encoding positive-sense single-stranded RNA encoding seven (7) non-structural and three (3) structural proteins. The dengue virus (DENV) contains four serotypes identified as DENV 1–4 [10,11]. The serotypes share similar genetic properties but with a different antigenic configuration, and infection with multiple serotypes increases the risk of severe complications from DENV. When mosquitoes bite humans, they inject DENV into the bloodstream, which then spreads to the epidermis and dermis, leading to the infection of young Langerhans cells (epidermal dendritic cells) and keratinocytes. The cells that have been infected migrate from the initial site to the lymph nodes. Monocytes and macrophages are attracted to the lymph nodes and thus become prone to infection [11]. Another route of virus dissemination is the release of exosomes from DENV-infected cells, which are responsible for viral transmission through cell–cell interaction [12,13]. Since viral infections induce exosome secretion, it is plausible to assume that DENV infection could also induce exosome release in dengue-infected patients. Dengue infection alters the composition of exosomes secreted by infected cells [12,13]. These exosomes carry the complete DENV genome and other proteins and transmit viral particles to healthy cells [12,13]. In addition, the exosomes released by DENV-infected cells contain LC3 II, an autophagy marker that defends the virus from neutralizing antibodies [13,14,15].

When a person who has not been previously infected with a flavivirus or immunized with a flavivirus vaccine acquires a dengue infection, IgM antibodies to the dengue virus can be detected 4–5 days after the onset of symptoms [10,15,16,17]. The symptoms are reliably identifiable for approximately 12 weeks. Detectable levels of serum IgG against dengue are observed by the end of the first week of illness [15,16,17,18,19]. These titers gradually increase and can remain detectable for several months or even for life. In cases of secondary dengue infection (when the host has been previously infected by dengue, another flavivirus, or vaccinated), antibody levels rise rapidly and can react broadly against many flaviviruses. The primary type of antibody is IgG, which is detected at high levels, even in the acute phase, and can persist for periods ranging from 10 months to a lifetime [15,16,17,18,19]. IgM levels during the early convalescent phase are lower in secondary infections than in primary infections and may even be undetectable in some cases, depending on the test used. 

In the pre-critical phase of the disease, there is a rapid decline in platelet count, accompanied by elevated hematocrit levels. DENV-specific antibodies play diverse roles, aiding in the clearance of the infection through various mechanisms [10,15,19,20,21]. This includes inhibiting the virus from binding to cell surface receptors or blocking viral entry post-binding. However, it is essential to note that the receptors to which DENVs bind present an opportunity for DENV-specific antibodies to potentially enhance viral entry, a phenomenon known as antibody-dependent enhancement (ADE) [20,21].

Upon infection of host cells, a range of pro-inflammatory, immunoregulatory, and antiviral cytokines are secreted. Dendritic cells (DCs) are recognized for producing type I interferons but may also release other pro-inflammatory molecules and cytokines. Studies have indicated that DENV-infected DCs secrete matrix metalloproteinases (MMP)-2 and 9, increasing endothelial monolayer permeability [22]. Various DENV proteins, such as NS4B and NS5, induce IL-8 synthesis by macrophages and endothelial cells. Furthermore, endothelial cells release IL-6, CXCL10, CXCL11, and RANTES, which elevate inflammation and vascular permeability, ultimately leading to in vivo plasma leakage [22,23].

**Figure 1:**
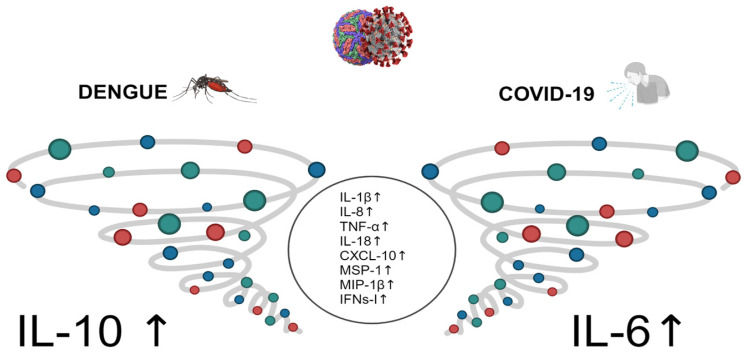
Comparison of the cytokine storm. The central circle represents a common pattern of cytokines observed in both dengue fever and SARS-CoV-2 infections. An uncontrolled inflammatory response leads to the concurrent release of elevated levels of various inflammatory cytokines, contributing to the pathogenesis associated with these infections. Numerous inflammatory cytokines and chemokines, including IL-6, IL-1β, IL-8, CCL8, CXCL9, CXCL16, MCP-1, and IP-10, as well as immunosuppressive cytokines, are notably elevated and correlate with the clinical severity of the disease. Although both viral infections, dengue and COVID-19, are characterized by a cytokine storm and multiorgan involvement, their pathogenesis, disease progression, and recovery processes differ significantly.

Published evidence indicates a shift in cytokine expression patterns during dengue infection, contributing to the observed “cytokine storm” (uncontrolled and excessive release of pro-inflammatory cytokines) in affected individuals. Figure 1 provides a visual representation of this phenomenon. The cytokine storm results in heightened vascular permeability and disruption of the coagulation cascade, leading to manifestations such as bleeding, serositis, and hypovolemic shock. The dengue virus’s non-structural protein 1 (NS1) has been implicated as the viral antigen responsible for mediating severe disease. NS1 initiates the complement cascade, leading to an excessive release of vasoactive anaphylatoxins, thereby inducing abnormal mast cell activation and histamine release, which in turn increases vascular permeability and causes endothelial dysfunction [10,15,24]. Furthermore, antibodies against NS1 (IgM and IgG) form complexes with membrane NS1 (mNS1) and soluble NS1 (sNS1), resulting in complement-dependent lysis of host cells and antibody-dependent cellular cytotoxicity (ADCC), ultimately damaging the endothelial layer and increasing vascular leakage [25]. Additionally, sNS1 interacts with TLR4, expressed on monocytes, macrophages, and endothelial cells, further exacerbating endothelial damage during DENV infection. Platelet activation and thrombocytopenia characterize the cytokine storm in DENV infection. This activation leads to the release of granular constituents. Patients with dengue exhibit signs of platelet activation, mitochondrial disruption, and activation of the caspase cascade of apoptosis, contributing to thrombocytopenia [24,25,26,27,28]. There is a clear association between ADE observed in laboratory experiments and clinical symptoms [26,27].

Preliminary studies have shown that NS1, released into patients’ blood, can stimulate immune cells via Toll-like receptor 4 (TLR4) and may cause endothelial leakage [29]. However, whether DENV NS1 can directly stimulate platelet activation or cause thrombocytopenia during DENV infection is unclear. Chao C.H. and coworkers [29] were the first to demonstrate that DENV, but not Zika virus, cell culture supernatant could induce P-selectin expression and phosphatidylserine (PS) exposure in human platelets and that both effects ceased when NS1 was removed from the DENV supernatant. Similar results were achieved using recombinant NS1 from all four DENV serotypes. This event suggests that overstimulation of lymphocytes is possible during dengue infection.

Recently, using the mouse model, Choi Y. and coworkers [30] have shown the importance of NKT cells in inducing Th1 polarity against dengue viral proteins. The process is regulated by CD1 presentation. A higher initial Th2 response, typified by a higher IgG4/IgG3 ratio, is involved with a worse outcome in the secondary infection. Thus, efficient antigen presentation is crucial for an effective immune response during dengue viral infection. This issue, however, may be controversial as it has been shown that in SARS-CoV-2 infection, the BCG vaccine protects lung disease in experimental mouse models [31]. No substantial evidence supports using the BCG vaccine to prevent human viral infection [32].

### 1.2. Severe Acute Respiratory Syndrome Coronavirus 2 (SARS-CoV-2)

Coronaviruses (CoVs) are divided into four different genera: α, β, γ, and δ. The α- and β-CoV genera infect mammals, while the γ- and δ-CoV infect birds. SARS-CoV-2 is a positive-sense, non-segmented, enveloped RNA (ribonucleic acid) virus belonging to the β-CoV. SARS-CoV-2 causes severe diseases among the human population, including respiratory, enteric, or systemic conditions of varying severity [33]. The virus originated in China, specifically in Wuhan city, Hubei province, in late 2019. The most common symptoms of SARS-CoV-2 infections are cough and fever, weakness, and loss of sense of taste or smell; other symptoms described are aches and pains, diarrhea, sore throat, and rash [33].

**Figure 2:**
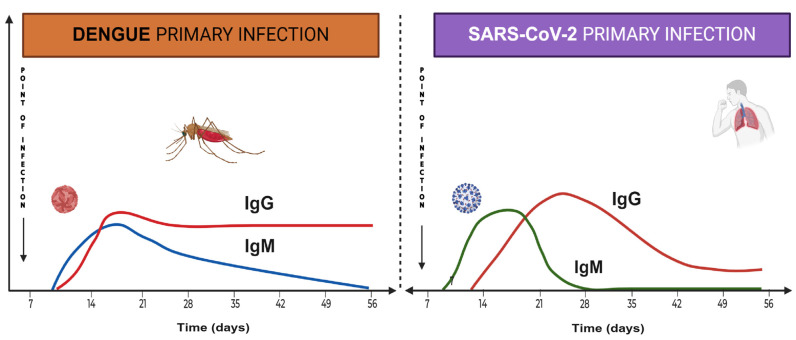
Antibody kinetics following initial viral infection. This figure illustrates the dynamics of antibody production during the first viral infections. IgM antibodies targeting dengue viral proteins exhibit longer persistence than those directed against SARS-CoV-2 proteins. IgG antibodies against SARS-CoV-2 demonstrate a decline after four weeks; conversely, antibody titers in dengue infections decrease at a significantly slower rate over time. This figure was created using BioRender^®^ software.

The body’s response to SARS-CoV-2 infection initially targets the N protein, but effective immunity relies on neutralizing antibodies against the virus’s S protein. Most COVID-19 patients develop antibodies around 7 days after contracting the virus. The average IgM and IgG antibody development times are 12 and 14 days, respectively. Xiaolong Yan et al. (2024) [34] found that IgG levels against SARS-CoV-2 decrease slowly over 12 months post-infection (Figure 2). Younger individuals tend to have higher IgG levels after infection, while older adults might have lower IgG levels and reduced effectiveness against severe COVID-19. On the other hand, Movsisyan, M. and colleagues (2024) [35] reported persistent seropositivity for both anti-SARS-CoV-2 (N) and anti-SARS-CoV-2 (S) among convalescent COVID-19 patients over a 21-month evaluation period. Clearly, the antibodies against the N protein suggest that these individuals were continuously exposed to the virus, maintaining the memory response and the titer of antibodies as described by Swadźba, J. and co-workers [36].

SARS-CoV-2, like DENV, has a positive-sense single-stranded RNA genome. DENV uses attachment factors such as glycosaminoglycans, immunomodulatory protein receptors, C-type lectins DC-SIGN, and mannose receptors to enter host cell receptors. On the other hand, SARS-CoV-2 mainly interacts with host cell receptors by using glycoproteins, specifically the angiotensin-converting enzyme-2 (ACE-2), although some other receptors may facilitate viral infection [37,38]. Both viruses can lead to a cytokine storm (Figure 1) and an inflammatory immune response [39,40]. This results in a higher concentration of antibodies and affects the coagulation system. Coinfections of DENV with SARS-CoV-2 can lead to organ failure, particularly in the cardiovascular, pulmonary, and Central Nervous System (CNS), and have a worse prognosis than single infections. Additionally, both viruses can infiltrate the CNS and cause severe cases [39,40].

Although COVID-19 was initially believed to be a highly inflammatory disease, new evidence suggests it can lead to significant immune suppression or deficiency in severe cases [37,38]. Activating specific immune cells can cause lung damage [37,38]. In contrast, decreased antiviral responses and dysregulation of other immune cells can create a state where the virus can replicate, making secondary infections more likely. Further investigation is needed to understand the vital role of the IL-6/STAT3 signaling pathway in this immune dysregulation [41,42,43].

Silvestre OM et al. [44] carried out a prospective study in Brazil involving 2351 subjects; the study suggests that individuals with a history of dengue infection have a lower mortality from COVID-19. However, they could not determine a causal association between previous dengue and immunity that improves the prognosis of SARS-CoV-2 infection. The protective response could be due to similarities in protein structure that promote antiviral response in these individuals.

## 2. Role of Cytokines in DENV and SARS-CoV-2 Infection

### 2.1. Interferon Signal Transduction and miRNA

Viral proteins of both dengue and SARS-CoV-2 have been found to interfere with IFN signaling [45,46]. In particular, the dengue viral protein NS2A inhibits IFN I early signaling, while NS4A, NS4B, and NS5 inhibit IFN signaling by blocking STAT1 and STAT2, and mainly NS5 seems to be involved in the degradation of STAT2 [45]. On the other hand, SARS-CoV-2 viral proteins affect IFN signal transduction using different proteins [46]. The N protein inhibits interferon regulatory factor (IRF) 3 and 9, the M protein and the nonstructural proteins (NSP) 3, 6, and 13 block IRF7 signaling, and NSP12, NSP13, and ORF6 block IFNω [46]. In summary, both IFN type I and type II signaling may be inhibited by both viruses. The therapeutic role of type I IFN in both viral infections is questionable unless treatment is applied at the beginning of the acute phase, which rarely occurs in the standard clinical environment. Another interesting issue is the role of miRNA in both viral infections impairing the host immune response [47].

Even though there have been several reports on the possible protagonist of miRNA in severe dengue and COVID-19, some of the reported miRNAs are not specific for antiviral response [48,49,50,51]. They are related to known chronic inflammatory diseases in which cytokines are essential [48,49,50,51]. This issue generates an interesting hypothesis on the possible therapeutic role of different RNA, miRNA, long circular, or noncoding RNA being delivered in the target tissues in acute viral infection to avoid cytokine storm [48,49,50,51]. Many questions can still be addressed on this issue, especially regarding blocking mediators involved in antiviral responses.

### 2.2. Cytokines in Viral Infection and Cytokine Storm

The production of cytokines upon viral infection has been widely studied, and IFNs type I are critical players in the initial state of the host response to the virus [45,46]. A delay in IFN responses may worsen the inflammatory response [52,53]. As Fenf E. and coworkers [54] describe, a decreased production of IFN is expected in aging. In addition, Cremoni M. [55] analyzed the relationship between low baseline IFN-γ as a predictor of hospitalization.

However, individuals may have subclinical inflammation related to senescence with specific characteristics (inflammaging) [56]. In this context, an impaired antiviral response is expected in these individuals. In SARS-CoV-2 infections, the patients with higher viral disease severity are the elderly and individuals with different comorbidities who may develop the cytokine storm [57]. Interestingly, well-controlled patients with chronic inflammatory diseases seem to have a lower risk of severe infection [58].

Recent research indicates that the inflammation linked to acute SARS-CoV-2 infection can endure for weeks or even months. Bonny T.S. et al. [53] discovered that plasma donors from convalescent COVID-19 patients exhibited higher levels of specific inflammation markers than controls. These markers included IFN-γ, certain interleukins (IL-12p70, IL-13, IL-1β, IL-2, IL-4, IL-5, and IL-33), and MCP-1, providing strong evidence of ongoing immune activation. Other studies have demonstrated persistent changes in immune indicators in recovered COVID-19 patients, regardless of disease severity [59].

Dayarathna S. et al. [60] determined the similarities and differences in cytokine and chemokine responses in these two infections, comparing responses in patients with different COVID-19 and acute dengue severity at various times of illness. Patients who experienced severe COVID-19 pneumonia and dengue hemorrhagic fever (DHF) had notably higher levels of IL-6, IL-10, and MIP3α during disease onset compared to those with mild disease. Individuals who succumbed to COVID-19 had the lowest levels of IFNγ in the early stages of the disease [60]. However, these levels remained stable in patients with DHF or dengue fever (DF) throughout the febrile and critical phases. Bhatt P. and coworkers [61] showed the kinetics of cytokine secretion for several days following the infection. Interestingly, the increase in IL-4, IL-10, and IL-13 matched patient recovery with a decrease in other proinflammatory cytokines. Thus, the weakened IFNγ response to SARS-CoV-2 and elevated levels of immunosuppressive IL-10 in both COVID-19 and dengue during the early phase of illness indicates an inadequate antiviral response that could contribute to the severity of the diseases.

In a recent review, Zhang J. [62] illustrated how IFN type I and II, IL-6, and HMGB1 are the key players in the cytokine release syndrome. This syndrome leads to changes in the adaptative immune cells that amplify or resolve the inflammatory response induced by viral infection. The difference refers to the signals that maintain the inflammatory response, i.e., viral proteins. The kinetics of cytokine responses differ between dengue and SARS-CoV-2 infection [62].

Even though cytokine storms can be generated by both viral infections and are related to severity, the key cytokines involved in dengue differ from those of SARS-CoV-2, as shown in Figure 1. Early induction of IFN signaling may facilitate viral clearance and an effective immune response. On the other hand, it is unclear if modulation of IFN I and II signaling by viral proteins may be related to the cytokine storm in both viral infections. Nonetheless, the effect of IFN type III (IFN λ) is protective when the IFN type I signal is decreased, as was shown with pegylated IFN λ in COVID patients [63,64] and in vitro in dengue infection [64]. More research is urgently needed in this area, particularly in analyzing multisystemic inflammatory syndrome (MIS) in the pediatric population.

## 3. Humoral Responses in Dengue and SARS-CoV2 Infections

### 3.1. Antibodies in Dengue and SARS-CoV-2 Infection

As described before, the dynamics of antibody response between the two viruses differ [16,34,35]. Figure 2 illustrates the kinetics of virus infection in both diseases. The titter of the IgM antibody markedly differs, being short-lived in SARS-CoV-2 infection and compared with dengue infection. Similarly, IgG titers decay after week four after SARS-CoV-2 infection compared to dengue. Since the presence of antibodies against the SARS-CoV-2 virus is short-lived, the possibility of reinfection increases. Notably, only a subset of antibodies against SARS-CoV-2 are neutralizing, suggesting a polyclonal stimulation of B cells. Therefore, specific T-memory cells are crucial to maintaining a proper antibody response.

**Figure 3:**
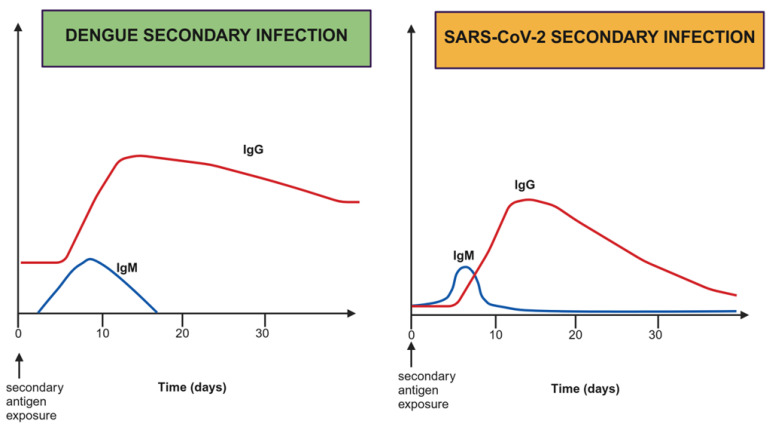
Differences in IgM and IgG kinetics in the second infection with dengue virus or SARS-CoV-2. On the left, the short time of IgM and the rapid and marked increase in IgG can be observed. In contrast to SARS-CoV-2 infection, IgM antibody timing differs, and IgG titers are relatively short lived. This figure was produced with the BioRender^®^ software.

Despite these efforts, many questions remain concerning how memory T and B cells are preserved in dengue infection [65,66,67]. In secondary infection, as illustrated in Figure 3, there is an increase in IgG production despite the high levels encountered before infection. It is also unclear how the kinetics of IgG titers are affected months after the second infection [67]. On the other hand, in SARS-CoV-2 infection, IgM titers increase slightly before IgG levels; basal IgG levels against the virus are low. Again, IgG is short lived, decreasing markedly after 21 days. More research is required to understand the role of memory cells and antibody dynamics in dengue pathophysiology and why they differ so much from SARS-CoV-2 infection.

**Figure 4:**
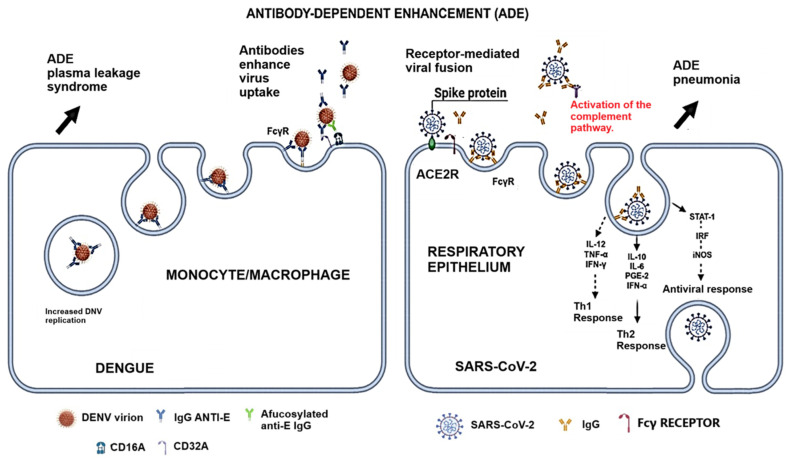
Antibody-dependent enhancement in dengue and SARS-CoV-2 infection. Antibody-dependent enhancement (ADE) occurs when antibody–virus complexes are internalized into cells through FcγRs, resulting in the infection of a more significant number of target cells. This process may lead to increased viral production. ADE in dengue infection has been proven. In SARS-CoV-2 infection, there are still controversies; however, several receptors and co-receptors for SARS-CoV-2 entrance to the cells have been proven and illustrated before [42]. This figure illustrates the proposed mechanism. This figure was made using the BioRender^®^ software.

Figure 4 illustrates the role of antibody-dependent enhancement in dengue and SARS-CoV-2 infections. The role of Fc receptors and virus uptake in macrophages has been demonstrated previously [20,21]. In SARS-CoV-2 infection, the ADE phenomenon differs from dengue since Fc-dependent infection of macrophages is inefficient; however, Fc-independent and cytokine-dependent induction of viral infection are observed in SARS-CoV-2 infection [68,69]. FcγRIIB (CD32B) was shown to be involved in virus internalization but requires a bivalent interaction [69]. Interestingly, Thomas S. and coworkers [70], using BHK cells expressing FcgRIIa, SARS-CoV-2, and MERS-CoV pseudoviruses (PVs), reported that samples from MERS-CoV-infected patients with low levels of neutralizing antibodies exhibited ADE against SARS-CoV-2. These results suggest that the ADE phenomenon may occur in SARS-CoV-2 infection and coinfection without neutralizing antibodies.

It is essential to add that even though the ACE2 receptor is critical for viral S protein binding and cell internalization, other receptors and coreceptors play a crucial role in SARS-CoV-2 infection, as previously reported [42].

Even though these viruses belong to different families, their genetic composition is similar, as they are both RNA viruses. Severe second infections mediated by ADE are observed in both diseases [65,66,67]. The presence of SARS-CoV-2 antibodies causing ADE in severe dengue has not been suggested before; there is a report on dengue infection and COVID-19 severity [5]. Additionally, COVID-19 and dengue co-infection have been linked to severe disease and fatal outcomes. After the COVID-19 pandemic, SARS-CoV-2 antibodies are much more common in the general population than in previous coronavirus epidemics. There is a possibility that these pre-existing COVID-19 antibodies could facilitate dengue virus entry into host cells, leading to increased viral load and severe disease [44,69,70,71]. More research is needed to clarify the phenomenon.

### 3.2. Autoantibodies After Dengue and SARS-CoV-2 Infection

Ghorai, T. et al. [72] demonstrated that antibodies produced during DENV infection can cross-react with several autoantigens in humans; these autoantibodies possibly contribute to clinical progression to severe dengue. Dengue was associated with an increased risk of autoimmune thyroid disease, uveitis, and autoimmune encephalomyelitis. Autoimmune encephalomyelitis is the most prevalent [73]. Since autoantibody screening is not standard after suffering from dengue infection, new guidelines should promote autoantibody screening after infection and follow-up. Chuang Y.C. and coworkers [74] reported molecular mimicry between dengue and coagulation factors to induce autoantibodies to block thrombin activity and enhance fibrinolysis. Similarly, after SARS-CoV-2 infection, several autoantibodies have been described. The generation of autoantibodies is related to a secondary immune deficiency in both cases. This secondary immune deficiency does not necessarily lead to autoimmune disease since most autoantibodies are short-lived, and an essential number of individuals are IgM and not IgG. The IgG encountered is not necessarily pathogenic.

The group of Jen Paul Casanova [75,76,77] has documented the presence of autoantibodies against cytokines that lead to immune deficiency. Autoantibodies against IFNα have been involved in the lack of proper antiviral response in several viral diseases, including dengue, SARS-CoV-2, and herpesvirus [78,79]. Nonetheless, there are still controversies on the role of these autoantibodies [80], and the loss of tolerance seems critical in the process [81].

As described previously [42], the presence of autoantibodies after SARS-CoV-2 infection has been described. These autoantibodies may induce a secondary immune deficiency, as described [42,81,82,83,84]. On the other hand, autoantibodies do not imply an autoimmune disease, as suggested [82]. Most of these autoantibodies are IgM, and some IgG levels, in most patients, decrease with time [42,83,84]. The presence of autoantibody titers may differ depending on SARS-CoV-2 variants [85] and vaccination [83,84]. A simple study showed that the incidence of antinuclear autoantibodies after SARS-CoV-2 infection was lower in the vaccinated individuals than those nonvaccinated [86]. Then, two possible explanations of the phenomenon may occur: molecular mimicry of antigens along with genetic predisposition or the polyclonal activation of B cells that, along with genetic predisposition and molecular mimicry, may be responsible for autoimmunity.

Autoantibodies against chemokines have also been reported in SARS-CoV-2 infection [87]. These autoantibodies block part of the antiviral immune response, but it isn’t easy to assume which autoantibody may be crucial for the antiviral response. As documented earlier, autoantibodies hamper IFN type I and II signaling and viral proteins [75,88,89,90]. It is unclear if both processes are involved in disease severity.

Table 1 sums up the autoantibodies detected in dengue and SARS-CoV-2 infection. Some of the autoantibodies are shared between both infections. It may be suggested that there is a reduced number of individuals with a subclinical autoimmune disease that, upon infection, there is a polyclonal activation of B lymphocytes, which are subsequently detected in preclinical tests. These autoantibodies may serve as a marker of a decreased efficiency of the antiviral response. Moreover, the presence of IgM autoantibodies and no increase in IgG antibodies with time suggests that B cell switching does not occur. This point should be considered for pharmacological intervention.

### 3.3. Thrombocytopenia in Dengue and SARS-CoV-2 Infections

Thrombocytopenia and platelet dysfunction are common in dengue and COVID-19 [69,70,96]. The coagulation and fibrinolytic pathways are activated during acute dengue infection, and endothelial dysfunction is seen in severe dengue. Traditionally recognized as responsible for homeostasis, platelets have become a critical factor in immunothrombotic complications associated with patients with COVID-19 [72,73,108]. At the same time, elevated levels of the soluble platelet selectin (sP-selectin), an indicator of platelet activation and endothelial injury, have been identified in patients with COVID-19 and associated with disease severity [109,110]. This complex connection underlines the crucial role of platelets and sP-selectin in coordinating thromboinflammation, vascular dysfunction, and disease progression in COVID-19 [109,110,111,112]. Platelet activation triggers the release of inflammatory mediators and increases platelet–leukocyte interactions, amplifying the systemic inflammatory response and aggravating endothelial injury. Furthermore, platelet-derived factors contribute to microvascular thrombosis, exacerbating tissue damage and organ dysfunction in severe COVID-19. Increased sP-selectin levels are disease severity and prognosis biomarkers, aiding risk stratification and early detection of patients most likely to suffer adverse outcomes [72,73,109,110,111,112]. Xu P. [113] has suggested several mechanisms as the cause of thrombocytopenia in COVID-19: (a) Direct infection of bone marrow cells by the virus and inhibition of platelet production. The cytokine storm destroys bone marrow progenitor cells and leads to reduced platelet production, (b) removal of platelets by the immune system, and (c) aggregation of platelets in the lungs.

Evidence suggests that the dengue virus may induce bone marrow hypoplasia during the acute phase of the disease [114,115]. Thrombocytopenia in dengue may be due to reduced bone marrow cell production or elevated peripheral platelet destruction and removal from peripheral blood. An increased mean platelet volume (MPV) indicates increased platelet destruction in patients. MPV is usually high or normal in dengue patients; therefore, excessive platelet destruction could be the main reason for thrombocytopenia in dengue patients. Cross-reactive anti-NS1, prM, and viral E protein antibodies against platelets, endothelial cells, or coagulant molecules may cause platelet disorders, endothelial cell injury, coagulation disorders, and macrophage activation. Platelet malfunction could increase the risk of vascular fragility, leading to hemorrhage and plasma leakage in DHF/DSS. There are few studies in dengue on platelets as effector immune cells.

## 4. Long-Term Alterations After SARS-CoV-2 Infection

Most people with SARS-CoV-2 infection develop lasting modifications in the immune response after natural infection [94,116]. Many studies have characterized these changes in both humoral and cellular responses. Regarding the humoral response, modest serological cross-reactivity between SARS-CoV-2 and DENV significantly affects the dengue diagnosis, as reported [43]. However, whether this could have any clinical impact on dengue cases is still unclear.

Guo L and colleagues [116] conducted a long-term study that included COVID-19 patients who had recovered and were discharged from Jinyintan Hospital in Wuhan, China. They followed up with the patients at six months, one year, and two years after their recovery, collecting blood samples each time. The participants were divided into two groups: those who had not been reinfected or vaccinated against SARS-CoV-2 and those who received one to three doses of an inactivated vaccine 1 to 2 years after recovering from the infection. The study evaluated the levels of IgG antibodies, neutralizing antibodies, memory B cells, and memory T cell responses against the original strain of the virus and the delta and omicron variants [116]. The findings indicated that memory T cell responses from the initial infection remained highly effective after two years. Given the increasing prevalence of new variants, the study suggests the need for vaccines that can enhance neutralizing antibodies and overall T-cell responses to newly emerged SARS-CoV-2 variants [116].

Patients with COVID-19 produce a wide range of autoantibodies that closely resemble the sequences of the highly mutated S protein of SARS-CoV-2 [117]. This is due in part to cell stress caused by viral infection. The antibodies are produced against various immune-regulating proteins, cytokines, chemokines, complement, certain intracellular cells, and specific viruses. Factors leading to the production of autoantibodies include changes in immune tolerance, increased cell death resulting from the non-apoptotic elimination of virus-infected cells, uncontrolled cytokine secretion (including TNFα, IFNβ, IL-6, IL-1β, IL-17, and IL-18), and abnormal expression of antigens following virus infection and inflammation [40,42,94,98,108]. Current research has revealed that even in COVID-19 patients with mild illness, acute SARS-CoV-2 infection has long-term impacts on the immune system [108,118]. Both humoral immunity and cellular immunity are affected.

Onofrio L.I. et al. [119] showed the difference in T-cell subpopulations in COVID-19 patients depending on waves or variants of the virus, generating an exciting analysis of antiviral immune response. In the first wave, moderate and severe COVID-19 patients showed significantly higher levels of granzyme B (GZMB) and expression of CD107a in CD8 cells and CD39 and PD-1 in conventional CD4+ T cells compared to healthy individuals [119]. In the second wave, there was a significant increase in the GZMB+ cells of moderate and severe COVID-19 patients. These patients also exhibited a decrease in the frequency of IL-2-producing T conv cells. Subpopulations of CD8 with low and high expression were shown to be important in SARS-CoV-2 infection. SARS-CoV-2-infected patients displayed fewer cells expressing low CD8 (CD8lo). These CD8lo cells secreted less TNF, IL-2, and IFN-γ than T cells with high CD8 expression (CD8hi). The frequency of CD8lo T cells increased with disease severity, suggesting its potential applicability as a marker for disease progression, and the index (CD8hi/CD8lo) was helpful in the clinical stratification and prediction of disease outcomes for patients [119].

CD8 cells are also important markers for dengue infection. Activated CD8 cells, HLADR+, and CD38 high T-cell subpopulations have been shown in dengue patients, especially during febrile response [120]. This cell population produces small amounts of IFNγ, and its response to a viral antigen is lower than that of the subpopulation with lower CD38 expression; however, antigen-independent activation of this cell population is normal [121,122,123,124]. On the other hand, CD4 and CD8 cells expressing CD38 were also observed in COVID-19 patients and related to severity, suggesting that the role of these cells in antiviral response is critical [123]. Vaccine-activated CD8 cells may be prevalent in SARS-CoV-2 viral infection [124]. On the other hand, IFNγ has been related to mild forms of dengue infection and SARS-CoV-2 infection [125]. Both cell populations, high and low IFNγ producers, can be valuable markers for SARS-CoV-2, dengue, and the common Zika infection in dengue-endemic areas. More research is required to understand the role of different cell subpopulations in viral infections.

During disease onset, patients who developed severe COVID-19 pneumonia and DHF had significantly higher levels of IL-6, IL-10, and MIP3α than those who developed mild disease [57]. The lowest levels of IFNγ in early disease were observed in those who succumbed to their disease by COVID-19. In contrast, these cytokine and chemokine levels remained unchanged in those with DHF or DF during the febrile and critical phases [57,125]. The low IFNγ response to SARS-CoV-2 and high levels of immunosuppressive IL-10 in COVID-19 and dengue during the early phase of illness indicate a poor antiviral reaction that could contribute to disease severity [57,125].

Severe COVID-19 patients exhibit a profound hypercoagulable state, and thrombotic complications are common. Excessive clotting has been observed in severely ill COVID-19 patients [126]. Lippi et al. [112] conducted an electronic search of Medline, Scopus, and Web of Science to select studies that provided data on platelet counts in patients with COVID-19. A meta-analysis was performed, calculating the weighted mean difference (WMD) of platelet counts in patients with COVID-19 with or without severe disease, and the odds ratio (OR) of thrombocytopenia was calculated for severe forms of COVID-19. The study found that a low platelet count is associated with an increased risk of severe disease and mortality in patients with COVID-19, similar to dengue disease [112]. It should serve as a clinical indicator of worsening disease during hospitalization.

Long COVID is a complication of SARS-CoV-2 infection, occurring in at least 10% of severe cases [127]. The causes are likely multiple and overlapping, including the persistence of SARS-CoV-2 in tissues, immune dysregulation, effects on microbiota, autoimmunity, blood clotting, and signaling abnormalities in the brainstem and vagus nerve [127,128]. Research is in the early stages, and while some theories have advanced, many questions remain unanswered. This area needs to be prioritized for further study.

## 5. Vaccines

### 5.1. Dengue Vaccines

The research and development of an accessible, safe, and effective vaccine against all four DENV serotypes is a breakthrough in the control of the disease and could contribute to achieving the WHO’s goal of reducing dengue morbidity and mortality. Dengvaxia (CYD-TDV) was the first quadrivalent DENV vaccine developed by Sanofi Pasteur to treat severe secondary dengue infection. It received its first marketing authorizations in 2015 and is currently approved for use in the US, EU, and some Asian and Latin American countries [129]. Dengvaxia is a live attenuated chimeric yellow fever–dengue-tetravalent dengue vaccine initially licensed by several endemic countries [129]. Unfortunately, the hope that a dengue vaccine was finally available quickly became a safety issue. In the third year of the Phase III clinical trial, younger subjects who received the vaccine and were not immune, experienced higher rates of hospitalization and severe dengue compared to unvaccinated subjects. Many hypotheses have been proposed to explain this, including the idea that imbalanced homotypic and heterotypic immunity among the four DENV types primed naïve (serostatus-negative) dengue vaccine recipients for ADE when exposed to their first natural infection. Sanofi sought an indication only for older children (9 years and older). Regulators forced the company to modify the vaccine label to indicate that only individuals previously infected with DENV should be vaccinated. A wave of protests emerged in the Philippines, as hundreds of thousands of children had already been immunized between the time of licensing and the discovery of this adverse event. Subsequently, the country suspended the licensing of the vaccine [130].

Takeda’s Denvax (TAK-003) was subsequently researched and developed as a live attenuated tetravalent dengue vaccine initially developed by the Division of Vector-Borne Diseases of the Centers for Disease Control and Prevention (CDC) [131]. TAK-003 contains a DENV-2 construct in which the pre-membrane (prM) and envelope (E) structural genes are replaced with chimeric DENV1, DENV3, and DENV4 viruses. Although TAK-003 trials are still in the large-scale evaluation stage, its phase 3 part 1 is effective against ‘virologically proven dengue’ in endemic areas such as Latin America and Asia among healthy children and adolescents aged 4–16 years [131].

The efficacy and safety profiles of TAK-003 have been demonstrated through a robust clinical trial program, including a 4.5-year Phase 3 study of over 20,000 children and adolescents living in eight dengue-endemic areas. The study was designed per World Health Organization (WHO) guidance for a second-generation dengue vaccine, and it considered the need to achieve high levels of subject retention and protocol compliance in endemic regions. Takeda’s vaccine, marketed as Qdenga^®^, is already approved in countries including Indonesia, Thailand, Argentina, and Brazil and was also licensed in the European Union in 2022. The Japanese company is in talks with the Indian pharmaceutical regulator for approval in the dengue-endemic country [131,132].

With no early manifestations of serious adverse effects, Qdenga^®^ is well tolerated and immunogenic against all four dengue serotypes, regardless of baseline serostatus, with a recent BLA priority review with the FDA [131,132]. In summary, both Dengvaxia^®^ and Qdenga^®^ are vaccines with great potential to change the prevention of DENV infection worldwide through a multimodal approach. Additionally, Qdenga^®^ can be used by travelers to endemic areas regardless of serological status [133,134].

### 5.2. COVID-19 Vaccines

COVID-19 vaccines have been shown to generate a lasting T-cell response [135] and generate an essential difference between infection and vaccination [136]. The strongest protection is derived from hybrid immunization [136]. The imprinting generated upon vaccination is particularly important for the mRNA vaccine [137].

A potential risk associated with the COVID-19 vaccines is antibody-dependent disease enhancement (ADE), where vaccine-induced antibody-mediated immune responses may lead to increased acquisition of SARS-CoV-2 or increased disease severity. Although ADE has not been clinically demonstrated with any of the COVID-19 vaccines, when neutralizing antibodies are suboptimal, COVID-19 severity has been reported to be increased [41].

An exciting report should be taken into account. Scully M et al. [138] presented the results of 23 patients who developed thrombosis and thrombocytopenia between 6 and 24 days after receiving the first ChAdOx1 nCoV-19 vaccine (AstraZeneca). Based on their clinical and laboratory features, they identified a new underlying mechanism and discussed therapeutic implications. Mild hematomas and petechiae were observed in some patients. Secondary cerebral hemorrhages were observed in some patients with cerebral venous thrombosis. One patient without thrombosis had clinically relevant hematomas but no other hemorrhagic manifestations. In all 23 patients, ELISA for anti-PF4 antibodies was performed on a sample obtained before administration of heparin-based therapy. While testing for heparin-induced thrombocytopenia (HIT) with the HemosIL AcuStar HIT, the IgG assay was negative in all nine patients tested, and ELISA for anti-PF4 antibodies was positive in 22 of the 23 patients. However, the syndrome described in the study presents a combination of clinical and laboratory features that constitute an exceptional case and has not been previously observed by any authors [138]. Ongoing data collection and studies may help to determine how the development of pathological platelet-activating anti-PF4 antibodies, unrelated to heparin use, might be associated with vaccination.

Ikewaki N. and coworkers [139] described the effect of glucans and their involvement in ADE in individuals vaccinated with COVID-19 vaccines. Interestingly, as proposed previously [42], glucans can enhance viral infection by facilitating virus cell entry or with the ADE complex. Future studies should address the importance of glucans in antiviral responses.

The research, development, approval, and widespread use of COVID-19 mRNA vaccines have constituted a significant asset in combatting the pandemic. However, it is imperative to assess changes in vaccination strategies now that substantial portions of the population have either been infected with SARS-CoV-2 or vaccinated, thereby acquiring some degree of immunity. The frequent administration of booster doses has prompted some experts to raise concerns regarding the potential for post-vaccination immune fatigue [140,141]. Furthermore, the increasing prevalence of mutations in the spike protein of emerging variants, combined with a gradual decline in the efficacy of mRNA vaccines, underscores the necessity to adapt vaccine formulations to include new viral targets [142]. For individuals predisposed to adverse cardiac effects, particularly during periods of heightened viral transmission, alternative vaccine types—such as viral vector or inactivated vaccines—should be considered.

A study conducted by Anderson E. and coworkers [143] analyzing SARS-CoV-2 vaccination in a cohort of patients with autoimmune diseases showed that vaccination did not aggravate the disease. In that cohort, the vaccine from Moderna (an mARN vaccine) had the lowest report of secondary effects. Therefore, it cannot be concluded that mRNA vaccine therapy predisposes people to the induction of autoimmune antibodies unless autoantibodies against the S protein are present during vaccination, inducing coagulopathy [144]. The presence of these autoantibodies is scarce and may be linked to genetic predisposition [144]. There is still room for improvement in this area.

## 6. Conclusions

The similarities in clinical manifestations between COVID-19 and dengue present challenges in diagnosis, particularly in regions where dengue is prevalent and resources are limited. Both SARS-CoV-2 and DENV can trigger immune cell activation, releasing proinflammatory cytokines, albeit with differences in the resulting cytokine storm. While antibody kinetics vary between the two infections, it is essential to note that antibody-dependent enhancement (ADE) is more frequently observed in dengue but should not be discounted in SARS-CoV-2 infection. Autoantibodies may play a significant role in the immunopathology of both infections. However, it remains unclear whether the production of autoantibodies results from a temporary polyclonal B cell activation since titers have been shown to decrease between 6 months and one year.

The potential for cross-reactivity of immune responses is concerning, particularly how pre-existing antibodies against DENV may impact COVID-19 through ADE. Co-infection with SARS-CoV-2 and dengue virus can result in more severe outcomes, leading to substantial morbidity and mortality. The overlapping clinical and laboratory features of both infections contribute to accurate diagnosis and complexity of management. Early diagnosis is crucial for mitigating the burden of these conditions. Despite differences in the modes of transmission (respiratory vs. mosquito-borne), it is imperative to investigate the immunopathogenesis similarities between the two infections to advance the development of more effective vaccines and treatments.

## Figures and Tables

**Table 1:** Autoantibodies detected in dengue and SARS-CoV-2 infection in severe, recovery, or chronic disease. The presence of an autoantibody is not necessarily related to an autoimmune disease. It may be an autoimmune phenomenon resulting from either the dengue virus or SARS-CoV-2 infection. However, it could modulate the course of the disease.

DENGUE	SARS-CoV-2
Anti-type-I-IFN autoantibodies [78]. Other anti-cytokine autoantibodies have not been analyzed.	Anti-type-I-IFN [75,79] and anti-type II IFN autoantibodies [88] in 5% of the population. Autoantibodies against chemokines [87].
Antibodies against platelets [91]. Antibodies against endothelial cells [61].	Autoantibodies against components of the cardiovascular system IgM and IgG [92]. Autoantibodies against ACE2 [93] and anti-heparin factor 4 [94].
Antibodies against receptors expressed on B cells TACI, BCMA, and BAFFR [95].	Autoantibodies against the lung antigen KCNRG [96] and IgA autoantibodies against pulmonary surfactant proteins B and C decrease mucus secretion [97].
IgG autoantibodies against several complement pathway components, such as Factor P and Complement C4 [98].	Autoantibodies detected: antinuclear antibodies, P-ANCA, C-ANA, anti-Ro52, anti-Ro60, rheumatoid factor, anti-citrullinated autoantibodies, SLE-associated Smith-D3 protein [99,100,101], and lupus anticoagulant [102]. Antibodies against MDA5 [103,104]
Autoantibodies against prothrombin were correlated with platelet counts in DHF patients [98].	Autoantibody against prothrombin [105].
Autoantibodies against KU(P70/P80), histone H-3 and H-4, M2, vitronectin, MPO, and Sm/RNP [98].	Antibodies against G-protein-coupled receptors and RAS [106].
Protective autoantibodies against viral non-structural protein 1 (NS1) [107].	Autoantibodies are against thyroid hormone [108].
